# Soybean Bioactive Peptides and Their Functional Properties

**DOI:** 10.3390/nu10091211

**Published:** 2018-09-01

**Authors:** Cynthia Chatterjee, Stephen Gleddie, Chao-Wu Xiao

**Affiliations:** 1 Nutrition Research Division, Food Directorate, Health Products and Food Branch, Health Canada, Banting Research Centre, 251 Sir Frederick Banting Drive, Ottawa, ON K1A 0K9, Canada; cynthia.chatterjee@canada.ca; 2 Ottawa Research & Development Centre, Central Experimental Farm, Agriculture and Agri-Food Canada, 960 Carling Avenue Building#21, Ottawa, ON K1A 0C6, Canada; steve.gleddie@agr.gc.ca; 3 Food and Nutrition Science Program, Department of Chemistry, Carleton University, 1125 Colonel By Drive, Ottawa, ON K1S 5B6, Canada

**Keywords:** soy protein, soy peptides, bioactives, functional property, chronic diseases, biomarkers

## Abstract

Soy consumption has been associated with many potential health benefits in reducing chronic diseases such as obesity, cardiovascular disease, insulin-resistance/type II diabetes, certain type of cancers, and immune disorders. These physiological functions have been attributed to soy proteins either as intact soy protein or more commonly as functional or bioactive peptides derived from soybean processing. These findings have led to the approval of a health claim in the USA regarding the ability of soy proteins in reducing the risk for coronary heart disease and the acceptance of a health claim in Canada that soy protein can help lower cholesterol levels. Using different approaches, many soy bioactive peptides that have a variety of physiological functions such as hypolipidemic, anti-hypertensive, and anti-cancer properties, and anti-inflammatory, antioxidant, and immunomodulatory effects have been identified. Some soy peptides like lunasin and soymorphins possess more than one of these properties and play a role in the prevention of multiple chronic diseases. Overall, progress has been made in understanding the functional and bioactive components of soy. However, more studies are required to further identify their target organs, and elucidate their biological mechanisms of action in order to be potentially used as functional foods or even therapeutics for the prevention or treatment of chronic diseases.

## 1. Introduction

Soybean (*Glycine max*) was cultivated in Asia for nearly 5000 years, first in China, then in Japan. It was introduced to Europe in the 18th century and then to the United States in the 19th century [1,2,3]. Soybean has been an important economic crop in the United States since the 1940s. Currently the United States is the leading soy producer and accounts for over 30% of the world’s production [3,4]. The popularity of soy foods or products has been rising in North America over the last decades, particularly after the U.S. Food and Drug Administration (FDA) approved the food health claim linking soy protein to the reduction of the risk for coronary heart disease in 1999 [4,5,6,7]. Soybean is a rich source of high-quality proteins containing all the essential amino acids found in animal proteins without cholesterol and with less saturated fat.

Epidemiological studies have associated soy consumption with potential benefits in reducing the risk for chronic diseases such as obesity, cardiovascular disease, insulin-resistance/type II diabetes, certain type of cancers, and immune disorders [3,5,6,8,9,10,11]. Soy proteins and their associated phytochemicals, mainly isoflavones, are believed to be responsible for these health benefits. However, the specific functional or bioactive component(s) in soy have not been identified nor their mechanism of action well understood. In recent years, research has focused more on biologically active or “bioactive” peptides derived from soybeans from processes mimicking gastrointestinal digestion. This paper summarizes the current knowledge about the soybean bioactive peptides and their roles in the modulation of physiological functions or prevention of chronic diseases.

## 2. Soy Composition and Major Bioactives

Soybeans are generally composed of ~35–40% protein, ~20% lipids, ~9% dietary fiber, and ~8.5% moisture based on the dry weight of mature raw seeds [3]. Their compositions vary with the variety and with the location and climate of the planting. The major soy components that have been shown to have biological activity include proteins or peptides, isoflavones, saponins, and protease inhibitors [8,12].

### 2.1. Soy Proteins and Subunits

The two major storage proteins, β-conglycinin (βCG, 7S) and glycinin (11S), comprise 80–90% of the total protein in soybean [5,6,13,14]. βCG is composed of α’, α, and β subunits, whereas glycinin is composed of acidic (A) and basic (B) subunits: A1aB2, A1bB1b, A2B1a, A3B4, and A5A4B3 [6,12]. Minor proteins in soybean include 2S, 9S, and 15S storage proteins; lectin; and Kunitz and Bowman–Birk (BBI) protease inhibitors [14]. Soy proteins with different ratios of βCG and glycinin are believed to have different nutritional and physiological effects [15,16]. Soy proteins with varying subunit compositions have also been shown to have significantly different functional properties in relation to quality, yield, and texture in tofu production [17]. Bioactive peptides are inactive when they are part of the parent protein sequence, but become activated upon release by enzymatic processing, gastrointestinal digestion, food processing, or fermentation [9,10,16]. They are usually 2 to 20 amino acids in length and are absorbed by the intestines into the blood circulation to exert systemic or local physiological effects in target tissues [9,11,16]. Maebuchi et al. has demonstrated that human intestinal absorption of 11S peptides resulted in significantly greater increase in venous blood amino acid concentrations than did 11S globulin or amino acid mixture when administered as a beverage [18]. This difference was particularly notable for aromatic and branched-chain amino acids, suggesting that hydrolyzed soy protein is faster and more efficiently absorbed in humans [18].

### 2.2. Soy Isoflavones

The other major bioactive compounds in soybeans are the isoflavones, which are associated with the soy proteins. Isoflavones are phytochemicals, often referred to as phytoestrogens because they structurally resemble 17β-estradiol, and can bind both estrogen receptors α and β (ERα/β), but have a higher affinity for ERβ [19,20,21,22]. They possess both estrogenic and anti-estrogenic properties as shown in cell culture and clinical studies. Most of the isoflavones are naturally present in soybeans as glycosides, genistin, diadzin, and glycetin. However, upon digestion or fermentation by β-glucosidases they are converted to the bioactive form, aglycones: genistein, diadzein, and glycetein [6,8]. Soy isoflavones have been linked with beneficial effects in preventing heart disease, diabetes, menopausal symptoms, osteoporosis, and prostate and breast cancers [23,24,25,26] in humans because of their hormonal and antioxidant properties [23,27]. The large variation in abundance of each isoflavone in soybeans and soy foods and their bioavailability results in inconsistent physiological functions found among different studies [3,8,23,25,26].

### 2.3. Soy Saponins

A minor bioactive component in soybeans are the saponins which are amphiphilic oleanane triterpenoid glycosides with polar sugar chains conjugated to a nonpolar pentacyclic ring [28]. It is suggested that saponins have anti-inflammatory, anti-carcinogenic, antimicrobial, and hepato- and cardio-protective effects [28]. The effect of saponins is not further discussed in this review, which mainly focuses on bioactive soy peptides.

## 3. Methods of Bioactive Peptide Production

Soy bioactive peptides are small protein fragments produced by enzymatic hydrolysis, fermentation, food processing, and gastrointestinal digestion of larger soybean proteins [11,29] and are associated with a multitude of beneficial metabolic effects [16]. The peptide production and composition by different methods are affected by the enzymes (in vitro enzymatic hydrolysis) or bacteria (in fermentation) used and also related to the type of soy proteins.

### 3.1. Gastrointestinal Digestion

In its simplest form, soy bioactive peptides are released upon ingestion and digestion of soybeans by acid and digestive enzymes from the stomach, small intestine, and pancreas such as pepsin, trypsin, chymotrypsin, and pancreatin. These small peptides are absorbed through the walls of the small intestine into the bloodstream where they can have systemic effects or target specific tissues [30,31,32].

### 3.2. In Vitro Enzymatic Hydrolysis

In vitro enzymatic hydrolysis is applied commercially in larger volumes, which can have better quality control and are more effective and stable in obtaining peptides with specific molecular weight and peptide profiles [16]. In vitro enzymatic hydrolysis can also utilize a combination of specific and nonspecific proteases such as pepsin, trypsin, chymotrypsin, papain, and peptidase to obtain peptides from digestion of soy proteins under their optimal pH and temperature conditions.

### 3.3. Food Processing

Bioactive peptides can be formed during food processing because of structural or chemical alterations. For example, pH modifications or chemical treatments may lead to the modification of amino acids, altering functional properties [33]. Improved functionality can lead to improvements in digestibility, protein or peptide enrichment, or reduction of trypsin inhibitor activity, which can arise from acylation, glycosylation, phosphorylation, reductive alkylation, succinylation, or lipophilization [33]. Common food processing techniques include heat treatment, pH modification, protein separation, ultra-high-pressure processing, and storage conditions [33,34].

### 3.4. Bacterial Fermentation

Traditionally, Asian countries like Korea, China, and Japan have been consuming fermented soybean foods such as soy sauce, soy paste, natto, tempeh, and miso for a long time [3]. Fermentation is an efficient and cost-effective method for generating bioactive peptides and food-grade hydrolyzed proteins through microbial activity or microbial enzymatic activity [16,34]. A large group of bacteria known as lactic acid bacteria found in the upper gastrointestinal tract are frequently used in fermentation to produce bioactive peptides [16]. However, fermentation may not fully hydrolyze soybean proteins with post-translational modification and complex tertiary structures. It is necessary to supply additional enzymes such as pronase, trypsin, and plasma proteases to produce smaller peptides with better bioactivity [16,34,35]. In addition, fermentation plays an important role in texture and flavor development [16].

## 4. Soy Bioactive Peptides and Their Properties

Over the last decade or so, the focus of soy research has shifted to the identification and characterization of bioactive peptides and their corresponding physiological functions. Numerous soy peptides with widespread beneficial physiological effects have been identified as shown in Table 1. These include lipid lowering (hypocholesterolemic, hypotriglyceridemic, anti-obesity) to anti-diabetic, anti-cancer, hypotensive, anti-inflammatory, and antioxidant in a variety of experimental models.

### 4.1. Hypolipidemic

The best studied bioactivity of soy peptides is their hypolipidemic property. Many soy peptides have been identified to lower cholesterol and triglycerides, and to suppress fat synthesis and storage in different experimental systems. LPYPR from the glycinin subunit of the soybean was one of the initial hypocholesterolemic peptides discovered by Yoshikawa et al. (2000). Administration of this peptide at a dose of 50 mg/kg of body weight without isoflavones for 2 days reduced both serum total and low-density lipoprotein (LDL) cholesterol in rats by ~25% [53]. Subsequent studies further showed that, more specifically, LPYP was hypocholesterolemic [84] and acting as a competitive inhibitor of 3-hydroxy-3-methylglutaryl CoA reductase (HMGR), the major rate-limiting enzyme in cholesterol biosynthesis [49]. This peptide increased LDL uptake in cultured liver cells through activating the LDL receptor (LDLR)–sterol regulatory element-binding protein 2 (SREBP2) pathway [36].

Two other cholesterol-lowering peptides derived from glycinin are IAVPGEVA and IAVPTGVA [50,51]. Similar to LPYP, these peptides were shown to inhibit HMGR activity in cultured HepG2 cells and promote LDL uptake via the LDLR–SREBP2 pathway [36,49]. Lammi et al. uncovered two hypocholesterolemic peptides—YVVNPDNDEN and YVVNPDNNEN—derived from soy βCG, which also modulate cholesterol by an identical mechanism [37]. Some other hypocholesterolemic soy peptides include lunasin (2S) peptides [29], SFGVAE [49], WGAPSL [12,16], LLPHH [34], RPLKPW [34], VAWWMY [85,86,87], and FVVNATSN [88].

Moreover, several hypotriglyceridemic di-peptides have been identified from soybean components. These are KA (from glycinin, βCG, trypsin inhibitor, and lipoxygenase), VK (from glycinin, trypsin inhibitor, and lipoxygenase), and SY (from glycinin, βCG, and lipoxygenase) [67]. βCG was shown to inhibit fatty acid synthesis in the liver leading to a reduction in serum triglycerides in rats [68]. Further studies have identified that the peptides KNPQLR, EITPEKNPQLR, and RKQEEDEDEEQQRE in this protein were able to suppress fatty acid synthase (FAS) activity [16,42]. βCG subunits demonstrated a better ability to reduce lipid levels in mouse 3T3-L1 adipocytes by embedding more active peptides in the cells than glycinin subunits [89], and to suppress lipid accumulation by downregulating lipoprotein lipase and FAS [90]. Peptides ILL, LLL, and VHVV derived from Flavourzyme^®^-treated soy protein isolate showed lipolysis-stimulating activity in 3T3-L1 mouse adipocytes [76,91].

### 4.2. Anti-Diabetic

Obesity and hyperlipidemia are often associated with insulin resistance and type II diabetes leading to the metabolic disease phenotype. Interestingly, many soy peptides with hypolipidemic function also possess anti-diabetic activity in different experimental models. For example, the hypocholesterolemic soy peptides LPYP, IAVPGEVA, and IAVPTGVA also improved glucose metabolism by increasing glucose uptake in cultured hepatic cells via glucose transporters (GLUT) 1 and 4 [36,92]. Further in vitro and in silico studies demonstrated that peptide IAVPTGVA was an efficient inhibitor of dipeptidyl peptidase IV (DPP-IV), a serine exopeptidase. DPP-IV is responsible for the hydrolysis of glucagon-like peptide and glucose-dependent insulinotropic polypeptide which are critical for maintaining glucose homeostasis [52]. Although the peptides YVVNPDNDEN and YVVNPDNNEN had similar hypocholesterolemic activity as IAVPTGVA, they were ineffective at inhibiting DPP-IV due to their longer peptide sequence and lack of Pro as the fourth N-terminal residue [52].

Both soy protein and isoflavones have been linked with improvements in diabetic rodent models [93,94,95] such as lowered serum glucose levels, increased insulin secretion, and reduced fasting plasma glucose. However, fermented soybean products containing soy peptides like natto and chungkookjang may be even better in the prevention of the onset of type II diabetes in human and mouse models [10,96,97,98]. Consumption of a diet containing soy protein (35% animal protein, 35% soy protein, and 30% other plant proteins) for 6 weeks by women aged 18–40 years (at week 24–28 of gestation) with gestational diabetes mellitus (*n* = 34) was associated with significant improvements in fasting plasma glucose, serum insulin levels, homeostasis model of assessment—insulin resistance, and quantitative insulin sensitivity check index compared with the control diet group consisting of 70% animal and 30% plant proteins (*n* = 34) [99]. Studies by Oliva et al. demonstrated that dyslipidemic insulin-resistant Wistar rats fed a sucrose-rich diet supplemented with soy protein had decreased hepatic triglyceride and cholesterol storage and steatosis, functional muscle glucose transporter GLUT4, and normalized glucose-6-phosphate and glycogen levels [100]. In spontaneously diabetic Goto-Kakikazi rats, consumption of βCG specifically improved muscle glucose uptake with higher plasma adiponectin, increased GLUT4 translocation, and phosphorylated adenosine monophosphate-activated protein kinase (AMPK) [101]. Similarly, soymorphin-5 (YPFVV), a soy-derived µ-opioid peptide derived from the β-subunit of the βCG lowered glucose and triglyceride levels in diabetic KKAy mice through activation of adiponectin and peroxisome proliferator-activated receptor α (PPARα) [43]. Roblet et al. utilized electrodialysis with an ultrafiltration membrane to isolate low-molecular-weight (300–500 Da) soy peptides from a complex soy mixture [102]. This peptide fraction improved glucose uptake in cultured rat muscle cells through activation of AMPK by phosphorylation [102]. Another study showed that the soybean peptide, Vglycin, is resistant to digestive enzymes and has anti-diabetic function in type II diabetic Wistar rats [66]. Vglycin comprises 37 amino acids with 6 half-cysteines that are part of 3 pairs of disulfide bonds. When administered to diabetic Wistar rats for 4 weeks, it normalized fasting glucose levels, increased insulin sensitivity, and restored insulin signaling and pancreatic function [66].

### 4.3. Anti-Hypertensive

High blood pressure or hypertension is another risk factor for coronary heart disease. Interestingly, antihypertensive peptides are the most commonly occurring and best studied bioactive peptides in foods [11,16]. Antihypertensive peptides function by blocking angiotensin-converting enzyme (ACE), which modulates the rennin–angiotensin system, thereby regulating blood pressure [11,34]. The dipeptidyl carboxypeptidase activity of ACE converts the decapeptide angiotensin I into the vasoconstricting octapeptide angiotensin II, resulting in increased blood pressure [11]. Traditional Asian fermented soybean foods such as soybean paste [77], soy sauce [103], natto [104], and tempeh [105] are rich in ACE inhibitory peptides [81,106]. Korean fermented soybean paste treated with chymotrypsin contains the hypotensive tripeptide, HHL [12,16], while soybean fermented with *Bacillus natto* or *Bacillus subtilis* was shown to contain two ACE inhibitor peptides, VAHINVGZK and YVWK [16,34]. Fermented soybean seasoning was shown to have a higher ACE inhibitory activity compared with soy sauce [82] and this was attributed to the peptides SY and GY, which decreased hypertension in salt-sensitive Dahl rats by suppressing the renin–angiotensin system and lowering serum aldosterone levels [83]. Okara, a soy pulp extract that is a by-product of tofu production, has been shown to have ACE inhibitory activity due to the presence of some small antihypertensive peptides [107].

The other antihypertensive soy peptides include PGTAVFK, IVF, LLF, LNF, LSW, LEF, YVVFK, IPPGVPYWT, PNNKPFQ, NWGPLV, and TPRVF [16,34]. Treatment of soy milk with the industrial protease PROTIN SD-NY10 produced FFYY, WHP, FVP, LHPGDAQR, IAV, VNP, LEPP, and WNPR peptides that had enhanced ACE inhibitory activity compared with regular soy milk [80]. In soy βCG, LAIPVNKP and LPHF were demonstrated to have ACE inhibitory activity, while glycinin was found to contain the ACE inhibitory peptides VLIVP, SPYP, and WL, and more specifically, the A4 and A5 subunits of glycinin comprised the antihypertensive peptide sequence of NWGPLV [9,12]. It has been revealed that some structural similarities exist among the bioactive peptides with blood pressure lowering properties. The presence of Pro or hydroxyl-Pro at the C-terminus made the peptides generally resistant to degradation by digestive enzymes, while Pro, Lys, or Arg were preferred at the C-terminus for ACE inhibitory potency [9,34]. It was also observed that dipeptides with a C-terminal Tyr had higher antihypertension effect than dipeptides with C-terminal Phe [9].

### 4.4. Anti-Cancer

Soy isoflavones have drawn much scrutiny over the years in terms of their role in cancer from both a promotion and prevention standpoint [3,6]. However, soy peptides have also been identified in different experimental systems to have anti-cancer properties [11,12,16,29,56]. Back in 2000, Kim et al. purified the hydrophobic peptide X-MLPSYSPY from defatted soy protein that arrested the cell cycle progression of murine lymphoma cells (P388D1) at G2/M phase [69]. Further studies have shown that most of the anti-cancer soy peptides belong to the minor 2S fraction of soybean proteins: lunasin and BBI [12,16,29,56,57,58]. The BBI is a low-molecular-weight protein, can inhibit trypsin and chymotrypsin activity, and has been considered as an anti-nutrient for a long time [57,108]. It has demonstrated anti-carcinogenicity in different species including humans and tissue types including colon, liver, esophagus, breast, prostrate, and was considered as an FDA Investigational Drug in 1992 [57,108]. In both Phases I and II human trials, the BBI promised to be a safe cancer chemopreventive agent that prevents and suppresses malignant transformation and carcinogenesis at doses from 800 to 2000 chymotrypsin inhibitor units [108,109]. The mechanism by which BBI exerts its anti-cancer activity involves apoptosis through reactive-oxygen-species-induced mitochondrial damage after proteasomal inhibition and anti-angiogenesis [110,111,112,113].

Lunasin (SKWQHQQDSCRKQKQGVNLTPCEKHIMEKIQGRGDDDDDDDDD) is another chemopreventive peptide that is closely associated with the BBI. It is 43 residues long with a C-terminal of 9 aspartic acid residues and cell adhesion motif, RGD that enables binding to non-acetylated H3 and H4 histones to prevent their acetylation, providing its anti-carcinogenic activity [16,34]. Park et al. showed that the BBI has a function of protecting lunasin from gastrointestinal degradation when soy protein is consumed orally [58]. Lunasin decreased skin tumor incidence in the SENCAR mice skin cancer model by ~70% when topically applied at a dose of 250 µg, and promoted colony suppression of mammalian cells induced by carcinogens and viral oncogenes E1A and RAS by 30–43% [11,12,114,115,116,117]. However, its effects on human breast cancer cell line MCF-7 appear to be inconsistent. Lunasin did not inhibit the growth rate of MCF-7 and mouse fibroblase NIH 3T3 cancer cells in vitro [117], whereas more recent study showed that lunasin induced apoptosis in MCF-7 cells by upregulation of tumor suppressor PTEN similar to the soy isoflavone genistein [56]. Lunasin is also able to inactivate the tumor suppressor proteins, Rb, p53, and pp32, and competes with the histone acetyltransferases in binding to the core deacetylated histones H3 and H4, and switching off the transcription, leading to arrest of the G1/S phase and causing apoptosis [29].

### 4.5. Antioxidant and Anti-Inflammatory

Carcinogenesis and cancer development depend in part on pro-inflammatory, pro-oxidant, and immunosuppressive mechanisms that lead to abnormal growth of tissue. Consequently, proteins and peptides with anti-cancer properties often also exhibit anti-inflammatory and antioxidant effects [29,70]. Soy protein with or without isoflavones was shown to reduce oxidative stress and have anti-inflammatory properties by inhibiting nuclear factor-kappa B (NF-κB) and blocking the secretion of pro-inflammatory cytokines in an oxidative-stress–inducible rat model, a hyperlipidemic mouse model, humans with end-stage renal disease, and healthy women over 70 years of age [70]. Soy milk digested with pepsin and pancreatin produced the bioactive peptides RQRK and VIK, which inhibited lipopolysaccharide-induced inflammation in murine macrophages. These hydrolysates inhibited the production of nitric oxide, interleukin (IL)-1β, nitric oxide synthase, and cyclooxygenase-2 [79].

Lunasin’s anti-cancer potential arises from its dual anti-oxidative and anti-inflammatory capacity [29]. As an anti-oxidant, lunasin was shown to inhibit 2,20-azino-bis(3-ethylbenzothiazoline-6-sulfonic acid) diammonium salt radical scavenger, reactive oxygen species production, and the secretion of pro-inflammatory cytokines (Tumor Necrosis Factor-α and IL-6) in mouse RAW 264.7 macrophages [29,118]. It acts as a potent peroxyl and superoxide scavenger, and can prevent glutathione peroxidase and catalase activities [119]. The RGD motif of lunasin was responsible for blocking inflammation in human macrophages by interacting with αVβ3 integrin through an Akt-mediating NF-κB pathway [120]. A trial in healthy men demonstrated that ingestion of 50 g of soy protein resulted in the absorption rate of ~4.5% of the total lunasin ingested [121].

### 4.6. Immunomodulatory

Closely associated with anti-cancer, anti-oxidant, and anti-inflammatory peptides are immunomodulatory peptides. Immunomodulatory peptides boost immune cell functions; for example, natural killer cell activity or cytokine regulation [16]. These peptides have been found in soy protein hydrolysates that are enzymatically digested [34]. The hydrolysates prepared from insoluble soy protein with alcalase had the greatest murine splenic lymphocyte proliferation and phagocytosis capability in peritoneal macrophages [16,122]. The peptides HCGAPA and GAPA from the glycinin component of soy protein hydrolysate stimulated phagocytosis [39,54]. The trypsin digests of soy proteins revealed that the sequence MITLAIPVNKPGR was able to stimulate phagocytosis in leukocytes [34,39]. This peptide is derived from the α’-subunit of βCG and was named soymetide and later soymetide-13 since Met at its N-terminus was essential for its activity [34,39]. Some of the C-terminus residues of soymetide-13 could be removed to form soymetide-9 (MITLAIPVN) which had the highest activity. Soymetide-4 (MITL) was the minimal sequence required for its activity [39]. In general, the soymetides had an affinity for the N-formyl-methionyl-leucyl-phenylalanine receptor despite not being formylated at the N-terminus Met [39,40].

### 4.7. Neuromodulatory

The β-subunit of βCG contains the sequence for human β-casophormin-4 (YPFV), an opioid peptide that has morphine-like activity. This has resulted in the discovery of three peptides with anxiolytic activities: soymorphin-5 (YPFVV), soymorphin-6 (YPFVVN), and soymorphin-7 (YPFVVNA) [44]. These peptides were selective for the µ opioid receptor and were shown to suppress food intake and small intestinal transit due to the coupling of the receptor to neurotransmitters in mice [45]. In addition, soymorphin-5 was shown to improve glucose and triglyceride levels in a KKAy diabetic mouse model by activating adiponectin and PPARα, and promoting β-oxidation and energy expenditure [43]. These peptides may not need to be absorbed into the blood circulation for their anxiolytic effects. The β-subunit of βCG also contains the peptide VRIRLLQRFNKRS (fragment 51–63) which suppressed food intake and gastric emptying in rats by stimulating a mediator of satiety, plasma cholecystokinin, through an extracellular calcium-sensing receptor [46,47,48].

## 5. Conclusions

Soybean is a promising source of peptides that have a wide range of biological activities such as hypolipidemic, anti-diabetic, anti-hypertensive, anti-cancer, antioxidant, anti-inflammatory, immunostimulatory, and neuromodulatory properties demonstrated in different models. Further studies are warranted for better understanding of their absorption, metabolism, and target tissues, as well as for elucidating their mechanisms of actions. A high quality of human trials will help in this regard as well as address the bioavailability of the peptides. Certain functions of the soy peptides such as the anxiolytic effects of soymorphins may not require their absorption into the blood circulation. However, anti-cancer or hypolipidemic peptides need to be bioavailable to pass through the small intestines into the bloodstream to reach their target tissues. More studies are needed to identify the quantity of the active soybean peptides released by different methods (for example, in vivo or in vitro digestions), and the impact of gender and age on the action or production of bioactive soybean peptides.

## Figures and Tables

**Table 1:** Soy bioactive peptides and their properties.

Soy Protein Source	Bioactive Peptide	Properties	Tested Model	Reference
βCG	YVVNPDNDEN	Hypocholesterolemic	HepG2 human liver cells	[36,37]
	YVVNPDNNEN			
	LAIPVNKP	ACE inhibition	In vitro ACE inhibitory activity assay	[12,38]
	LPHF			
βCG (α’-subunit)	Soymetide-13: MITLAIPVNKPGR	Immunostimulating	Male ICR mice and fMLP receptor binding assay; Phagocytosis assay; Anti-alopecia in neonatal rat model	[12,34,39]
	Soymetide-9: MITLAIPVN			[34,39,40,41]
	Soymetide-4: MITL			
	KNPQLR; EITPEKNPQLR; RKQEEDEDEEQQRE	FAS inhibitor	FAS inhibition studies; 3T3-L1mouse adipocyte	[16,42]
βCG (β-subunit)	Soymorphin-5: YPFVV	Anti-diabetic Triglyceride-lowering Immunostimulating Suppress feeding and intestinal transit	Guinea pig ileum assay opioid activity; Diabetic KKA^y^ mice	[39,43,44,45]
	Soymorphin-6: YPFVVN		Elevated Plus-Maze Test in Male ddY Mice	
	Soymorphin-7: YPFVVNA		Male BALB/c and ddY mice	
	VRIRLLQRFNKRS	Appetite suppressant	Male BALB/c and ddY mice; Male Sprague-Dawley rat; Mouse intestinal STC-1 cells	[9,39,46,47,48]
Glycinin	IAVPGEVA	Hypocholesterolemic Anti-diabetic	HMGR activity assay; HepG2 human liver cells; DPP-IV activity assay	[9,36,49,50]
	IAVPTGVA			[36,50,51,52]
	LPYP			[36,37,39,49,53]
	VLIVP	ACE inhibition	ACE inhibitory assay	[12]
	SPYP			
	WL			
	SFGVAE	Hypocholesterolemic	HMGR activity	[49]
	HCQRPR	Phagocytosis stimulatory peptide	Macrophages; Human polymorphonuclear leukocytes; C3H/He mouse	[16,39,54]
	QRPR			
Glycinin (A4 and A5)	LPYPR	Hypocholesterolemic	Mice at dose of 50 mg/kg for 2 days; HMGR activity assay;	[9,16,34,39,53]
	NWGPLV	ACE inhibition	Spontaneously Hypertensive model rats	[9,12,55]
Lunasin	SKWQHQQDSCRKQKQ GVNLTPCEKHIMEKIQ GRGDDDDDDDDD	Antioxidative Anti-inflammatory Anti-cancer Hypocholesterolemic	Suppression of skin papilloma development in SENCAR mice by acting as an antimitotic agent; Synergistically works with cytokines (IL-12 or IL-2) to improve the tumoricidal activity of natural killer cells in in vitro and in vivo tumor models; TEN-mediated apoptosis of MCF-7 breast cancer cells; Inhibits production of HMGR and increases LDLR expression; Antioxidant in Caco-2 cells; Inhibit ROS generation in HepG2 cells; Inhibit proinflammatory cascades in THP-1 macrophages	[11,12,16,29,56]
Bowman-Birk Inhibitor		Anti-cancer Proteinase inhibition Chemoprevention	50% reduction in the frequency of chromosomal abnormalities and sister chromatid exchange in blood syndrome patients; Shrink precancerous lesions in the mouth that lead to oral cancer called leukoplakia in humans in Phase I and II clinical trials; Reduction in the level of serum PSA in males with benign prostatic hyperplasia; Blocks the generation of ROS in prostate cancer cells (BRF-55T, 267B1/Ki-ras, LNCaP, and PC-3 cells); protected Balb c/3T3 cells (clones A 31) exposed to UVC irradiation and reduced transformation;	[57,58,59,60,61,62,63,64,65]
	Vglycin	Anti-diabetic	Normalize fasting glucose and restore pancreatic function in Type 2 diabetic Wistar rats	[66]
Glycinin, βCG-α, βCG-α’, βCG-β, Trypsin Inhibitor & Lipoxygenase	KA	Triglyceride-lowering	HepG2 cells; Male Otsuka Long-Evans Tokushima fatty rats; Male Wistar rats	[67,68]
Glycinin, Trypsin Inhibitor & Lipoxygenase	VK			
Glycinin, βCG-α, βCG-α’, βCG-β & Lipoxygenase	SY			
Defatted soy protein	X-MLPSYSPY	Anti-cancer	Arrest P388D1 mouse monocyte macrophages at G2/M phase to block cell cycle progression	[16,34,69]
Soy protein	YVVFK; IPPGVPYWT; PNNKPFQ; NWGPLV; TPRVF	Hypotensive	Spontaneously hypertensive rats	
		Anti-inflammatory	Postmenopausal women; ApoE knockout mice	[70,71,72,73,74]
	WGAPSL; VAWWMY; FVVNATSN	Hypocholesterolemic	Rats; HepG2 cells	[12,16,39]
Soybean	PGTAVFK	Hypotensive	IC_50_ = 26.5 µM	[16,34]
	IVF; LLF; LNF; LSW; LEF	ACE inhibition	ACE inhibitory activity assay and UPLC-MS/MS	[75]
Flavouzyme^®^-treated soy protein isolate	ILL; LLL; VHVV	Lipolysis	3T3-L1 mouse adipocytes	[76]
Chymotrypsin Korean fermented soybean paste	HHL	Hypotensive	Spontaneously hypertensive rats	[77]
Genetically modified soybean protein	LLPHH; RPLKPW	Antioxidative; Antihypertensive		[34]
Black soybean protein	IQN	Adipogenesis inhibition	3T3-L1 mouse adipocytes	[16,78]
Soymilk	RQRK; VIK	Anti-inflammatory	RAW 264.7 mouse macrophages	[79]
Protease (PROTIN SD-NY10) treated soy milk	FFYY; WHP; FVP; LHPGDAQR; IAV; VNP; LEPP; WNPR	ACE inhibition	ACE inhibitory activity assay	[39,80]
Fermented soybean, Bacillus natto or subtilis	VAHINVGK		ACE inhibitory activity assay and simulated gastrointestinal digestion	[81]
	YVWK			
Fermented soybean seasoning	SY GY		Spontaneously hypertensive rats	[82,83]

## Abbreviations

ACE	angiotensin-converting enzyme
Akt	protein kinase B
AMPK	adenosine monophosphate-activated protein kinase
ApoE	apolipoprotein E
BBI	Bowman–Birk inhibitor
DPP-IV	dipeptidyl peptidase IV
ER	estrogen receptor
FAS	fatty acid synthase
fMLP	N-formylmethionyl-leucyl-phenylalanine
FDA	Food and Drug Administration
ICR	Institute of Cancer Research
IL	Interleukin
GLUT	glucose transporters
HMGR	3-hydroxy-3-methylglutaryl-CoA reductase
LDL	low-density lipoprotein
LDLR	LDL receptor
NF-κB	nuclear factor kappa B
PPARα	peroxisome proliferator-activated receptor α
PSA	Prostate specific antigen
ROS	Reactive oxygen species
SREBP2	sterol regulatory element-binding protein 2
UPLC-MS/MS	Ultra performance liquid chromatography-tandem mass spectrometry
UVC	Ultraviolet C
